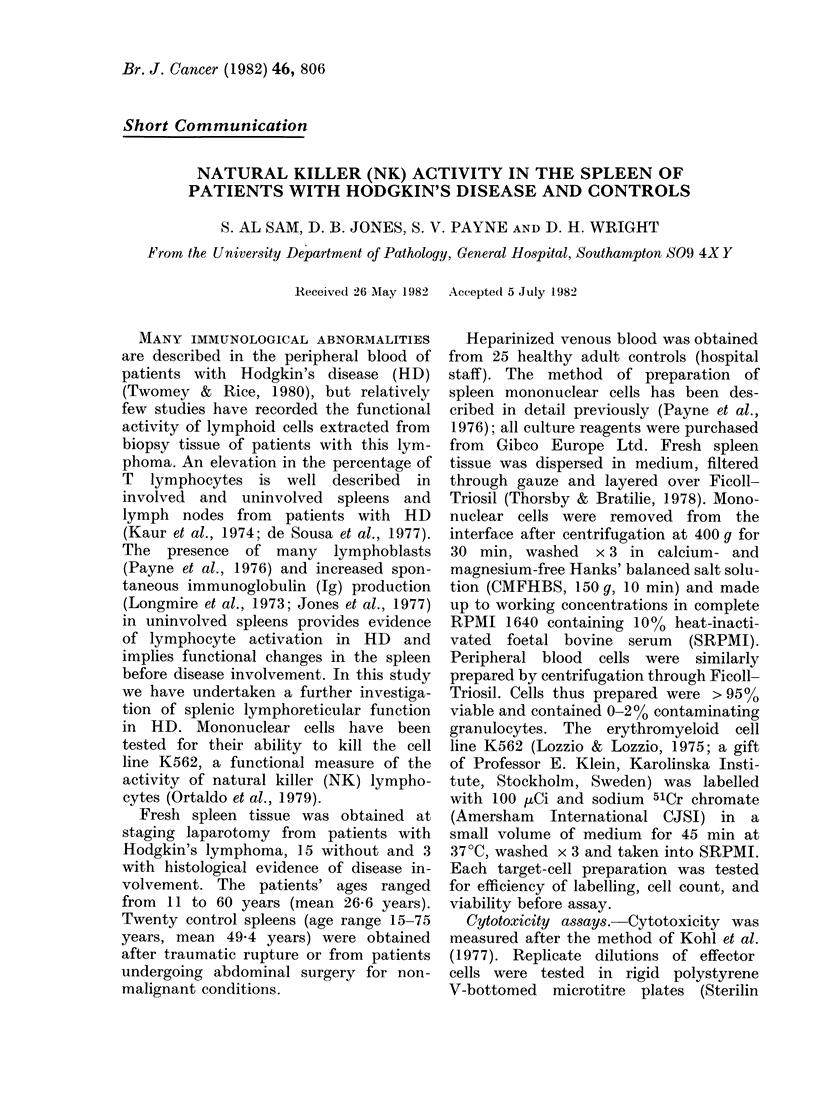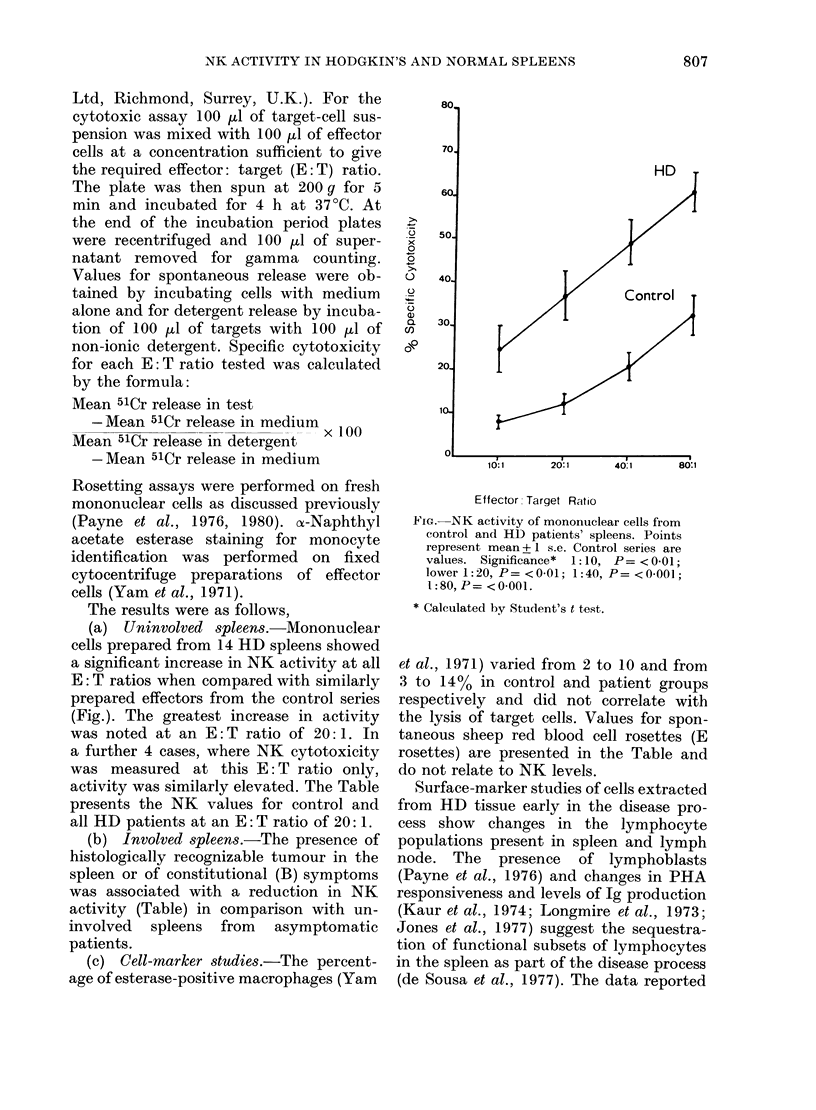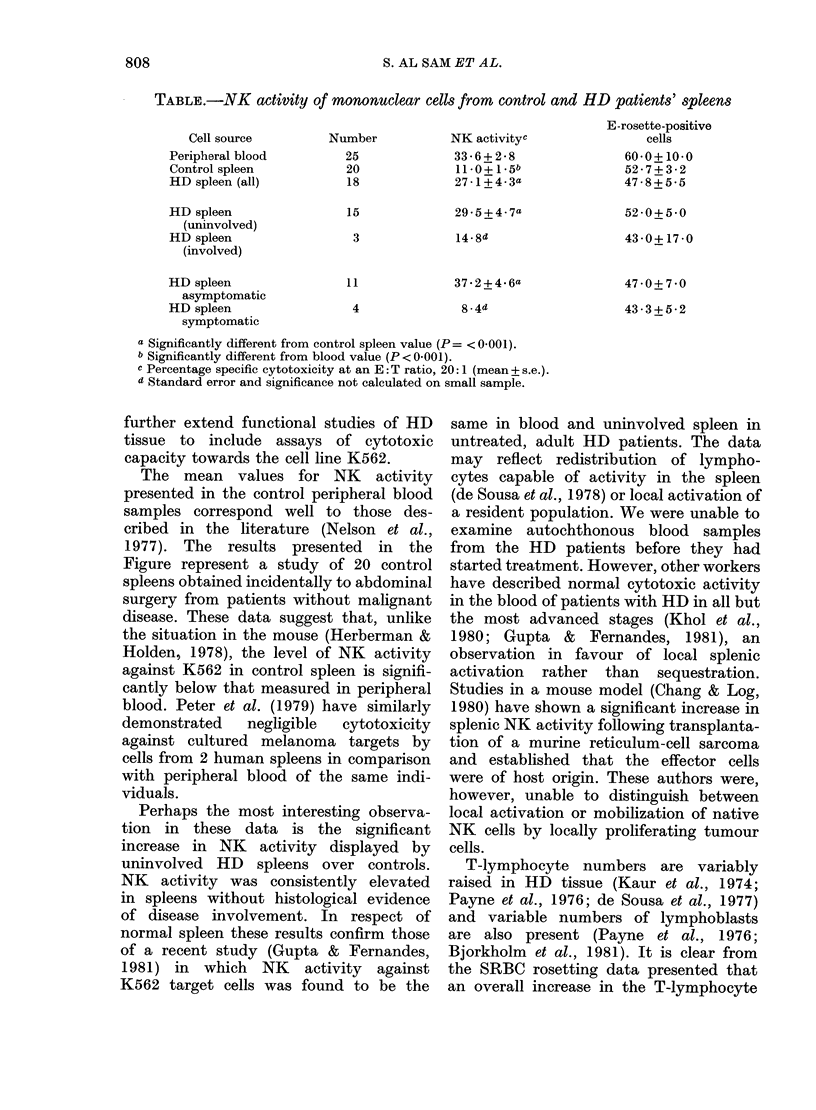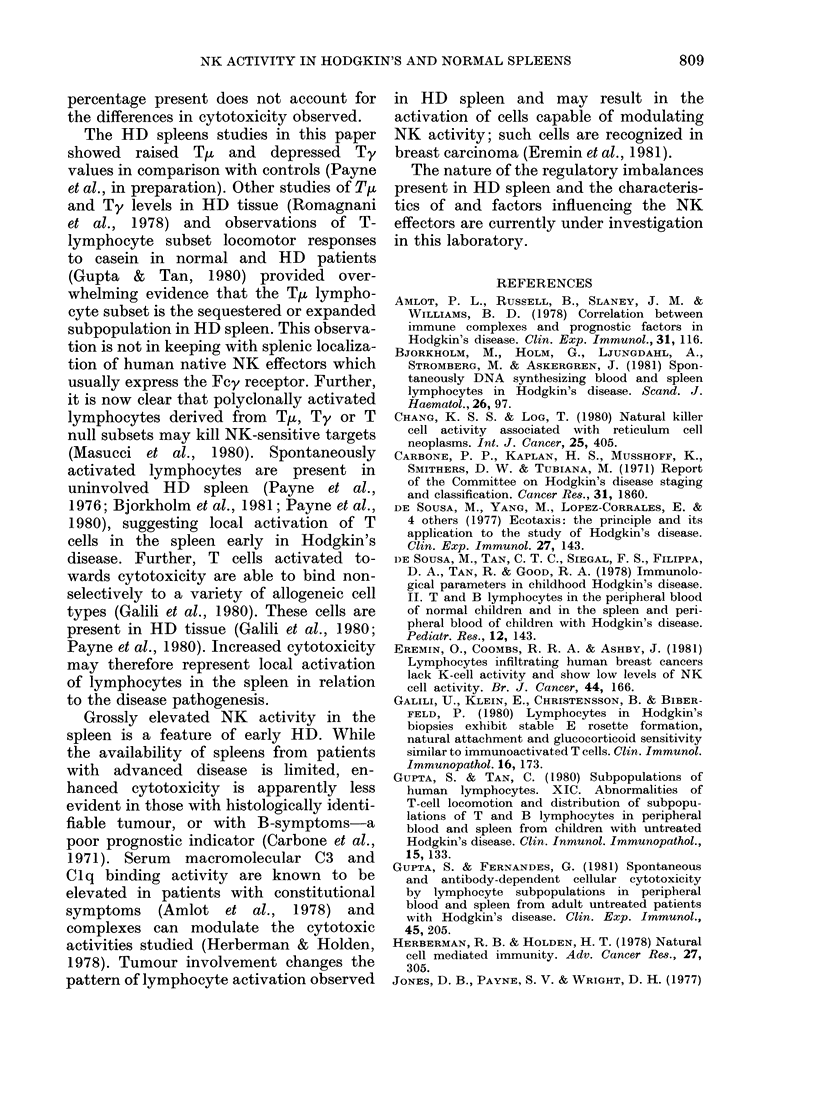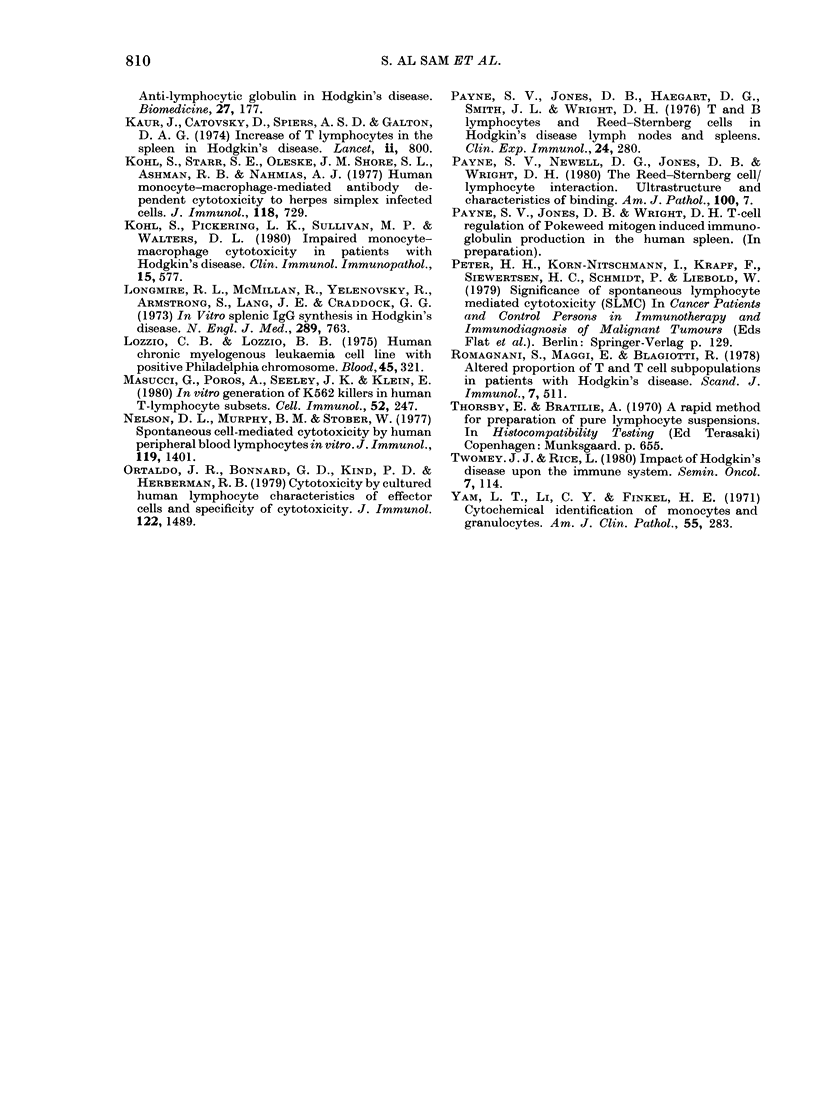# Natural killer (NK) activity in the spleen of patients with Hodgkin's disease and controls.

**DOI:** 10.1038/bjc.1982.274

**Published:** 1982-11

**Authors:** S. Al Sam, D. B. Jones, S. V. Payne, D. H. Wright


*Br. J. Cancer* (1982) **46**, 806

***Short Communication***

### NATURAL KILLER (NK) ACTIVITY IN THE SPLEEN OF PATIENTS WITH HODGKIN'S DISEASE AND CONTROLS

S. AL SAM, D. B. JONES, S. V. PAYNE AND D. H. WRIGHT

*From the University Department of Pathology, General Hospital, Southampton SO9 4XY*

Received 26 May 1982 Accepted 5 July 1982

MANY IMMUNOLOGICAL ABNORMALITIES are described in the peripheral blood of patients with Hodgkin's disease (HD) (Twomey & Rice, 1980), but relatively few studies have recorded the functional activity of lymphoid cells extracted from biopsy tissue of patients with this lymphoma. An elevation in the percentage of T lymphocytes is well described in involved and uninvolved spleens and lymph nodes from patients with HD (Kaur *et al.*, 1974; de Sousa *et al.*, 1977). The presence of many lymphoblasts (Payne *et al.*, 1976) and increased spontaneous immunoglobulin (Ig) production (Longmire *et al.*, 1973; Jones *et al.*, 1977) in uninvolved spleens provides evidence of lymphocyte activation in HD and implies functional changes in the spleen before disease involvement. In this study we have undertaken a further investigation of splenic lymphoreticular function in HD. Mononuclear cells have been tested for their ability to kill the cell line K562, a functional measure of the activity of natural killer (NK) lymphocytes (Ortaldo *et al.*, 1979).

Fresh spleen tissue was obtained at staging laparotomy from patients with Hodgkin's lymphoma, 15 without and 3 with histological evidence of disease involvement. The patients' ages ranged from 11 to 60 years (mean 26·6 years). Twenty control spleens (age range 15–75 years, mean 49·4 years) were obtained after traumatic rupture or from patients undergoing abdominal surgery for non-malignant conditions.

Heparinized venous blood was obtained from 25 healthy adult controls (hospital staff). The method of preparation of spleen mononuclear cells has been described in detail previously (Payne *et al.*, 1976); all culture reagents were purchased from Gibco Europe Ltd. Fresh spleen tissue was dispersed in medium, filtered through gauze and layered over Ficoll–Triosil (Thorsby & Bratilie, 1978). Mononuclear cells were removed from the interface after centrifugation at 400 *g* for 30 min, washed ×3 in calcium- and magnesium-free Hanks' balanced salt solution (CMFHBS, 150 *g*, 10 min) and made up to working concentrations in complete RPMI 1640 containing 10% heat-inactivated foetal bovine serum (SRPMI). Peripheral blood cells were similarly prepared by centrifugation through Ficoll–Triosil. Cells thus prepared were >95% viable and contained 0–2% contaminating granulocytes. The erythromyeloid cell line K562 (Lozzio & Lozzio, 1975; a gift of Professor E. Klein, Karolinska Institute, Stockholm, Sweden) was labelled with 100 μCi and sodium $^{51}$Cr chromate (Amersham International CJSI) in a small volume of medium for 45 min at 37°C, washed ×3 and taken into SRPMI. Each target-cell preparation was tested for efficiency of labelling, cell count, and viability before assay.

*Cytotoxicity assays.*—Cytotoxicity was measured after the method of Kohl *et al.* (1977). Replicate dilutions of effector cells were tested in rigid polystyrene V-bottomed microtitre plates (Sterilin

Ltd, Richmond, Surrey, U.K.). For the cytotoxic assay 100 $\mu$l of target-cell suspension was mixed with 100 $\mu$l of effector cells at a concentration sufficient to give the required effector: target (E:T) ratio. The plate was then spun at 200 *g* for 5 min and incubated for 4 h at 37°C. At the end of the incubation period plates were recentrifuged and 100 $\mu$l of supernatant removed for gamma counting. Values for spontaneous release were obtained by incubating cells with medium alone and for detergent release by incubation of 100 $\mu$l of targets with 100 $\mu$l of non-ionic detergent. Specific cytotoxicity for each E:T ratio tested was calculated by the formula:

$$\frac{\text{Mean } ^{51}\text{Cr release in test} - \text{Mean } ^{51}\text{Cr release in medium}}{\text{Mean } ^{51}\text{Cr release in detergent} - \text{Mean } ^{51}\text{Cr release in medium}} \times 100$$

Rosetting assays were performed on fresh mononuclear cells as discussed previously (Payne *et al.*, 1976, 1980). $\alpha$-Naphthyl acetate esterase staining for monocyte identification was performed on fixed cytocentrifuge preparations of effector cells (Yam *et al.*, 1971).

The results were as follows,

(a) *Uninvolved spleens.*—Mononuclear cells prepared from 14 HD spleens showed a significant increase in NK activity at all E:T ratios when compared with similarly prepared effectors from the control series (Fig.). The greatest increase in activity was noted at an E:T ratio of 20:1. In a further 4 cases, where NK cytotoxicity was measured at this E:T ratio only, activity was similarly elevated. The Table presents the NK values for control and all HD patients at an E:T ratio of 20:1.

(b) *Involved spleens.*—The presence of histologically recognizable tumour in the spleen or of constitutional (B) symptoms was associated with a reduction in NK activity (Table) in comparison with uninvolved spleens from asymptomatic patients.

(c) *Cell-marker studies.*—The percentage of esterase-positive macrophages (Yam

FIG.—NK activity of mononuclear cells from control and HD patients' spleens. Points represent mean ± 1 s.e. Control series are values. Significance* 1:10, $P = <0{\cdot}01$; lower 1:20, $P = <0{\cdot}01$; 1:40, $P = <0{\cdot}001$; 1:80, $P = <0{\cdot}001$.

* Calculated by Student's *t* test.

*et al.*, 1971) varied from 2 to 10 and from 3 to 14% in control and patient groups respectively and did not correlate with the lysis of target cells. Values for spontaneous sheep red blood cell rosettes (E rosettes) are presented in the Table and do not relate to NK levels.

Surface-marker studies of cells extracted from HD tissue early in the disease process show changes in the lymphocyte populations present in spleen and lymph node. The presence of lymphoblasts (Payne *et al.*, 1976) and changes in PHA responsiveness and levels of Ig production (Kaur *et al.*, 1974; Longmire *et al.*, 1973; Jones *et al.*, 1977) suggest the sequestration of functional subsets of lymphocytes in the spleen as part of the disease process (de Sousa *et al.*, 1977). The data reported

TABLE.—*NK activity of mononuclear cells from control and HD patients' spleens*

| Cell source | Number | NK activity[c] | E-rosette-positive cells |
|---|---|---|---|
| Peripheral blood | 25 | 33·6±2·8 | 60·0±10·0 |
| Control spleen | 20 | 11·0±1·5[b] | 52·7±3·2 |
| HD spleen (all) | 18 | 27·1±4·3[a] | 47·8±5·5 |
| HD spleen (uninvolved) | 15 | 29·5±4·7[a] | 52·0±5·0 |
| HD spleen (involved) | 3 | 14·8[d] | 43·0±17·0 |
| HD spleen asymptomatic | 11 | 37·2±4·6[a] | 47·0±7·0 |
| HD spleen symptomatic | 4 | 8·4[d] | 43·3±5·2 |

[a] Significantly different from control spleen value ($P = < 0{\cdot}001$).
[b] Significantly different from blood value ($P < 0{\cdot}001$).
[c] Percentage specific cytotoxicity at an E:T ratio, 20:1 (mean ± s.e.).
[d] Standard error and significance not calculated on small sample.

further extend functional studies of HD tissue to include assays of cytotoxic capacity towards the cell line K562.

The mean values for NK activity presented in the control peripheral blood samples correspond well to those described in the literature (Nelson *et al.*, 1977). The results presented in the Figure represent a study of 20 control spleens obtained incidentally to abdominal surgery from patients without malignant disease. These data suggest that, unlike the situation in the mouse (Herberman & Holden, 1978), the level of NK activity against K562 in control spleen is significantly below that measured in peripheral blood. Peter *et al.* (1979) have similarly demonstrated negligible cytotoxicity against cultured melanoma targets by cells from 2 human spleens in comparison with peripheral blood of the same individuals.

Perhaps the most interesting observation in these data is the significant increase in NK activity displayed by uninvolved HD spleens over controls. NK activity was consistently elevated in spleens without histological evidence of disease involvement. In respect of normal spleen these results confirm those of a recent study (Gupta & Fernandes, 1981) in which NK activity against K562 target cells was found to be the same in blood and uninvolved spleen in untreated, adult HD patients. The data may reflect redistribution of lymphocytes capable of activity in the spleen (de Sousa *et al.*, 1978) or local activation of a resident population. We were unable to examine autochthonous blood samples from the HD patients before they had started treatment. However, other workers have described normal cytotoxic activity in the blood of patients with HD in all but the most advanced stages (Khol *et al.*, 1980; Gupta & Fernandes, 1981), an observation in favour of local splenic activation rather than sequestration. Studies in a mouse model (Chang & Log, 1980) have shown a significant increase in splenic NK activity following transplantation of a murine reticulum-cell sarcoma and established that the effector cells were of host origin. These authors were, however, unable to distinguish between local activation or mobilization of native NK cells by locally proliferating tumour cells.

T-lymphocyte numbers are variably raised in HD tissue (Kaur *et al.*, 1974; Payne *et al.*, 1976; de Sousa *et al.*, 1977) and variable numbers of lymphoblasts are also present (Payne *et al.*, 1976; Bjorkholm *et al.*, 1981). It is clear from the SRBC rosetting data presented that an overall increase in the T-lymphocyte percentage present does not account for the differences in cytotoxicity observed.

The HD spleens studies in this paper showed raised $T\mu$ and depressed $T\gamma$ values in comparison with controls (Payne *et al.*, in preparation). Other studies of $T\mu$ and $T\gamma$ levels in HD tissue (Romagnani *et al.*, 1978) and observations of T-lymphocyte subset locomotor responses to casein in normal and HD patients (Gupta & Tan, 1980) provided overwhelming evidence that the $T\mu$ lymphocyte subset is the sequestered or expanded subpopulation in HD spleen. This observation is not in keeping with splenic localization of human native NK effectors which usually express the $Fc\gamma$ receptor. Further, it is now clear that polyclonally activated lymphocytes derived from $T\mu$, $T\gamma$ or T null subsets may kill NK-sensitive targets (Masucci *et al.*, 1980). Spontaneously activated lymphocytes are present in uninvolved HD spleen (Payne *et al.*, 1976; Bjorkholm *et al.*, 1981; Payne *et al.*, 1980), suggesting local activation of T cells in the spleen early in Hodgkin's disease. Further, T cells activated towards cytotoxicity are able to bind nonselectively to a variety of allogeneic cell types (Galili *et al.*, 1980). These cells are present in HD tissue (Galili *et al.*, 1980; Payne *et al.*, 1980). Increased cytotoxicity may therefore represent local activation of lymphocytes in the spleen in relation to the disease pathogenesis.

Grossly elevated NK activity in the spleen is a feature of early HD. While the availability of spleens from patients with advanced disease is limited, enhanced cytotoxicity is apparently less evident in those with histologically identifiable tumour, or with B-symptoms—a poor prognostic indicator (Carbone *et al.*, 1971). Serum macromolecular C3 and Clq binding activity are known to be elevated in patients with constitutional symptoms (Amlot *et al.*, 1978) and complexes can modulate the cytotoxic activities studied (Herberman & Holden, 1978). Tumour involvement changes the pattern of lymphocyte activation observed in HD spleen and may result in the activation of cells capable of modulating NK activity; such cells are recognized in breast carcinoma (Eremin *et al.*, 1981).

The nature of the regulatory imbalances present in HD spleen and the characteristics of and factors influencing the NK effectors are currently under investigation in this laboratory.

### REFERENCES

AMLOT, P. L., RUSSELL, B., SLANEY, J. M. & WILLIAMS, B. D. (1978) Correlation between immune complexes and prognostic factors in Hodgkin's disease. *Clin. Exp. Immunol.*, **31**, 116.

BJORKHOLM, M., HOLM, G., LJUNGDAHL, A., STROMBERG, M. & ASKERGREN, J. (1981) Spontaneously DNA synthesizing blood and spleen lymphocytes in Hodgkin's disease. *Scand. J. Haematol.*, **26**, 97.

CHANG, K. S. S. & LOG, T. (1980) Natural killer cell activity associated with reticulum cell neoplasms. *Int. J. Cancer*, **25**, 405.

CARBONE, P. P., KAPLAN, H. S., MUSSHOFF, K., SMITHERS, D. W. & TUBIANA, M. (1971) Report of the Committee on Hodgkin's disease staging and classification. *Cancer Res.*, **31**, 1860.

DE SOUSA, M., YANG, M., LOPEZ-CORRALES, E. & 4 others (1977) Ecotaxis: the principle and its application to the study of Hodgkin's disease. *Clin. Exp. Immunol.* **27**, 143.

DE SOUSA, M., TAN, C. T. C., SIEGAL, F. S., FILIPPA, D. A., TAN, R. & GOOD, R. A. (1978) Immunological parameters in childhood Hodgkin's disease. II. T and B lymphocytes in the peripheral blood of normal children and in the spleen and peripheral blood of children with Hodgkin's disease. *Pediatr. Res.*, **12**, 143.

EREMIN, O., COOMBS, R. R. A. & ASHBY, J. (1981) Lymphocytes infiltrating human breast cancers lack K-cell activity and show low levels of NK cell activity. *Br. J. Cancer*, **44**, 166.

GALILI, U., KLEIN, E., CHRISTENSSON, B. & BIBERFELD, P. (1980) Lymphocytes in Hodgkin's biopsies exhibit stable E rosette formation, natural attachment and glucocorticoid sensitivity similar to immunoactivated T cells. *Clin. Immunol. Immunopathol.* **16**, 173.

GUPTA, S. & TAN, C. (1980) Subpopulations of human lymphocytes. XIC. Abnormalities of T-cell locomotion and distribution of subpopulations of T and B lymphocytes in peripheral blood and spleen from children with untreated Hodgkin's disease. *Clin. Inmunol. Immunopathol.*, **15**, 133.

GUPTA, S. & FERNANDES, G. (1981) Spontaneous and antibody-dependent cellular cytotoxicity by lymphocyte subpopulations in peripheral blood and spleen from adult untreated patients with Hodgkin's disease. *Clin. Exp. Immunol.*, **45**, 205.

HERBERMAN, R. B. & HOLDEN, H. T. (1978) Natural cell mediated immunity. *Adv. Cancer Res.*, **27**, 305.

JONES, D. B., PAYNE, S. V. & WRIGHT, D. H. (1977)

Anti-lymphocytic globulin in Hodgkin's disease. *Biomedicine*, **27**, 177.

KAUR, J., CATOVSKY, D., SPIERS, A. S. D. & GALTON, D. A. G. (1974) Increase of T lymphocytes in the spleen in Hodgkin's disease. *Lancet*, **ii**, 800.

KOHL, S., STARR, S. E., OLESKE, J. M. SHORE, S. L., ASHMAN, R. B. & NAHMIAS, A. J. (1977) Human monocyte–macrophage-mediated antibody dependent cytotoxicity to herpes simplex infected cells. *J. Immunol.*, **118**, 729.

KOHL, S., PICKERING, L. K., SULLIVAN, M. P. & WALTERS, D. L. (1980) Impaired monocyte–macrophage cytotoxicity in patients with Hodgkin's disease. *Clin. Immunol. Immunopathol.*, **15**, 577.

LONGMIRE, R. L., MCMILLAN, R., YELENOVSKY, R., ARMSTRONG, S., LANG, J. E. & CRADDOCK, G. G. (1973) *In Vitro* splenic IgG synthesis in Hodgkin's disease. *N. Engl. J. Med.*, **289**, 763.

LOZZIO, C. B. & LOZZIO, B. B. (1975) Human chronic myelogenous leukaemia cell line with positive Philadelphia chromosome. *Blood*, **45**, 321.

MASUCCI, G., POROS, A., SEELEY, J. K. & KLEIN, E. (1980) *In vitro* generation of K562 killers in human T-lymphocyte subsets. *Cell. Immunol.*, **52**, 247.

NELSON, D. L., MURPHY, B. M. & STOBER, W. (1977) Spontaneous cell-mediated cytotoxicity by human peripheral blood lymphocytes *in vitro*. *J. Immunol.*, **119**, 1401.

ORTALDO, J. R., BONNARD, G. D., KIND, P. D. & HERBERMAN, R. B. (1979) Cytotoxicity by cultured human lymphocyte characteristics of effector cells and specificity of cytotoxicity. *J. Immunol.* **122**, 1489.

PAYNE, S. V., JONES, D. B., HAEGART, D. G., SMITH, J. L. & WRIGHT, D. H. (1976) T and B lymphocytes and Reed–Sternberg cells in Hodgkin's disease lymph nodes and spleens. *Clin. Exp. Immunol.*, **24**, 280.

PAYNE, S. V., NEWELL, D. G., JONES, D. B. & WRIGHT, D. H. (1980) The Reed–Sternberg cell/lymphocyte interaction. Ultrastructure and characteristics of binding. *Am. J. Pathol.*, **100**, 7.

PAYNE, S. V., JONES, D. B. & WRIGHT, D. H. T-cell regulation of Pokeweed mitogen induced immunoglobulin production in the human spleen. (In preparation).

PETER, H. H., KORN-NITSCHMANN, I., KRAPF, F., SIEWERTSEN, H. C., SCHMIDT, P. & LIEBOLD, W. (1979) Significance of spontaneous lymphocyte mediated cytotoxicity (SLMC) In *Cancer Patients and Control Persons in Immunotherapy and Immunodiagnosis of Malignant Tumours* (Eds Flat *et al.*). Berlin: Springer-Verlag p. 129.

ROMAGNANI, S., MAGGI, E. & BLAGIOTTI, R. (1978) Altered proportion of T and T cell subpopulations in patients with Hodgkin's disease. *Scand. J. Immunol.*, **7**, 511.

THORSBY, E. & BRATILIE, A. (1970) A rapid method for preparation of pure lymphocyte suspensions. In *Histocompatibility Testing* (Ed Terasaki) Copenhagen: Munksgaard. p. 655.

TWOMEY. J. J. & RICE, L. (1980) Impact of Hodgkin's disease upon the immune system. *Semin. Oncol.* **7**, 114.

YAM, L. T., LI, C. Y. & FINKEL, H. E. (1971) Cytochemical identification of monocytes and granulocytes. *Am. J. Clin. Pathol.*, **55**, 283.